# Lactylation: a metabolic-epigenetic bridge in diabetic kidney disease and a therapeutic target for TCM

**DOI:** 10.1186/s13020-026-01361-9

**Published:** 2026-03-06

**Authors:** Wen Zhang, Xiangdong Zhu, Yan Zhang, Jiahao Yang, Xiaobo Sun, Jianfeng Wang, Bin Zhang

**Affiliations:** 1 Henan University of Chinese Medicine, Zhengzhou, China; 2 Institute of Medicinal Plant Development, Peking Union Medical College and Chinese Academy of Medical Sciences, Beijing, China; 3 College of Traditional Chinese Medicine, Ningxia Medical University and Key Laboratory of Ningxia Minority Medicine Modernization Ministry of Education, Yinchuan, 750004 Ningxia Hui Autonomous Region People’s Republic of China; 4 Longhua Hospital, Affiliated to Shanghai University of Traditional Chinese Medicine, Shanghai, China; 5 Department of Pediatrics, Shimane University Faculty of Medicine, 89-1 Enya-Cho, Izumo, Shimane 693-8501 Japan

**Keywords:** Lactylation, DKD, Epigenetic regulation, Fibrosis

## Abstract

Lactate-derived lactylation, as an emerging epigenetic mechanism driven by lactic acid metabolism, plays a pivotal bridging role in diabetic kidney disease (DKD), linking metabolic dysregulation to pathological gene expression. Research indicates that lactacidosis exacerbates glomerular injury by promoting mesangial cell activation and podocyte damage, whilst simultaneously disrupting mitochondrial function in the tubules and activating pro-fibrotic pathways, thereby establishing a vicious cycle of metabolic reprogramming and fibrosis. Furthermore, lactate accumulation and lactylation modulate immune cell function, intensifying inflammatory responses. Owing to their multi-targeted properties, natural compounds from traditional Chinese medicine and traditional Chinese medicine compound formulations offer promising intervention strategies for this pathway. Specific agents such as Berberine, baicalin, ginsenoside compound K, and cordycepin have been demonstrated to mitigate renal injury by directly or indirectly reducing lactate production and modulating lactylation levels, thereby suppressing downstream inflammatory and fibrotic responses. This review systematically elucidates the pivotal role of the lactate-lactylation axis in the pathogenesis of DKD, highlighting traditional Chinese medicine's unique potential for precisely regulating this metabolic-epigenetic nexus. It offers innovative insights and strategies for the integrated management of DKD.

## Introduction

DKD is the primary cause of end-stage renal disease globally, posing a serious threat to human health and imposing a substantial socioeconomic burden. Recent epidemiological data indicate that approximately 20–40% of the world's nearly 589 million people with diabetes are projected to develop DKD, highlighting the urgent need for effective therapeutic strategies [1, 2]. The pathogenesis of DKD involves multifactorial mechanisms, including haemodynamic abnormalities, metabolic dysregulation, oxidative stress, and chronic inflammation [3–5]. In recent years, epigenetic regulation has emerged as a central mechanism linking these pathological processes, mediating persistent alterations in gene expression during DKD progression [6, 7]. Whilst the functions of classical modifications such as DNA methylation and histone acetylation/methylation have been extensively elucidated, the emergence of lactylation modifications signifies a paradigm shift—revealing the epigenetic mechanisms by which core metabolic by-products directly regulate gene transcription [8, 9].

A landmark study in 2019 systematically established lactidation as a genuine post-translational modification in mammalian cells, confirming that lactate produced via glycolysis directly regulates chromatin activity and gene expression by covalently modifying histone lysine residues [10]. This discovery positions lactylation as a fundamental metabolic sensor. In diabetic kidneys, extensive metabolic shift towards glycolysis leads to significant lactate accumulation, thereby driving excessive lactylation and its downstream effects [11]. Although initially studied in cancer contexts, the functional significance of lactylation has rapidly expanded to non-malignant pathologies characterised by inflammatory and fibrotic pathology, such as diabetic nephropathy [12]. A pivotal advancement lies in recent studies beginning to unravel specific mechanisms: histone lactylation drives transcription of pro-fibrotic genes like TGF-β1 in renal tubules, while lactylation of metabolic enzymes impairs mitochondrial function, forming a self-reinforcing cycle of injury [13, 14]. However, a systematic review is urgently needed to integrate how lactylation coordinates injury processes across distinct renal cell types and organelles.

Current first-line therapies primarily comprise angiotensin-converting enzyme inhibitors and sodium-glucose cotransporter 2 inhibitors, which though capable of delaying the progression of diabetic nephropathy, cannot halt its development. This underscores the necessity for therapeutic strategies targeting its fundamental mechanisms [1]. The multifaceted nature of lactylation renders it an immensely attractive yet complex therapeutic target, where traditional Chinese medicine demonstrates unique potential. Transcending conventional multi-target therapeutic approaches, recent studies indicate that specific herbal constituents such as berberine and astragaloside IV can directly modulate the lactate-lactylation axis [15]. This occurs either by inhibiting key drivers like lactate dehydrogenase (LDHA) or by regulating delactylating enzymes such as SIRT2 [16, 17]. This capacity for precise intervention within metabolic epigenetic networks positions TCM as a sophisticated strategy to disrupt the core pathophysiological mechanisms of diabetic nephropathy. Consequently, this review aims to consolidate key evidence establishing lactacidosis as a central driver of diabetic nephropathy while rigorously evaluating TCM's substantial potential for targeting this novel axis, thereby providing a novel conceptual framework for integrated interventions.

## Literature search strategy and study selection

This review was designed as a mechanism-oriented narrative review focusing on the emerging role of lactylation and lactate metabolism in DKD. To enhance transparency and methodological rigor, the processes of literature identification, screening, and selection were conducted in accordance with the core principles of the PRISMA 2020 statement, although a quantitative systematic review or meta-analysis was not intended.

A comprehensive literature search was conducted in PubMed, Web of Science, and China National Knowledge Infrastructure (CNKI) databases for studies published up to March 2025. Both Medical Subject Headings (MeSH) terms and free-text keywords were used. The main search terms included combinations of:

1. “Lactylation” OR “histone lactylation” OR “lactate metabolism”;
2. “Diabetic kidney disease” OR “diabetic nephropathy”;
3. “Traditional Chinese medicine”, “Chinese herbal medicine”, or “natural compounds”.

Studies were considered eligible if they met at least one of the following criteria:

1. Investigated lactate metabolism or lactylation-related mechanisms in DKD or diabetic complications;
2. Explored glycolysis-related pathways (e.g., LDHA, HIF-1α) or metabolic–epigenetic crosstalk relevant to renal cells or kidney injury;
3. Evaluated traditional Chinese medicine formulas or bioactive compounds with demonstrated effects on DKD pathology and/or lactate-related metabolic pathways.

Studies were excluded if they:

1. Were unrelated to kidney disease or diabetic complications;
2. Focused solely on lactylation in cancer or immune diseases without mechanistic relevance to DKD;
3. Lacked experimental or mechanistic evidence, such as purely descriptive reports.

Duplicate records were removed prior to screening. Titles and abstracts were first screened for relevance, followed by full-text evaluation of potentially eligible studies. Data extraction focused on experimental models, key molecular targets, involvement of lactate metabolism or epigenetic regulation, and implications for DKD pathogenesis. Notably, although direct experimental evidence linking traditional Chinese medicine to lactylation regulation in DKD is currently limited, studies demonstrating modulation of lactate metabolism or glycolytic pathways were retained, as they provide biological plausibility and hypothesis-generating value.

## Lactate metabolism and lactylation modifications: a new axis of epigenetic regulation

In diabetic kidneys, a widespread metabolic shift towards glycolysis accompanies mitochondrial dysfunction, ultimately leading to pathological lactic acid accumulation. Oxidative stress and chronic inflammation further exacerbate this metabolic disorder, impairing lactate dehydrogenase B (LDHB) function in pancreatic β-cells. This triggers a lactic acid-pyruvate metabolic imbalance and reduces lactate clearance capacity [18]. Within the renal microenvironment, persistent hyperglycaemia drives glycolytic activation, markedly increasing lactate production in both tubular and glomerular regions [16, 17]. Concurrently, mitochondrial tricarboxylic acid cycle dysfunction in proximal tubular cells further restricts lactate oxidation, while glycolysis in glomerular endothelial cells generates additional lactate, thereby establishing a self-perpetuating cycle of ‘lactate overload—enhanced lactate production—exacerbated renal injury’[16–18].

The molecular mechanisms regulating histone lactylation involve complex enzymatic and non-enzymatic pathways. At the enzymatic level, histone lactylation is primarily mediated by the p300/CREB-binding protein family, which utilises lactyl-CoA to catalyse lactylation of histone H3 at lysine 18 (H3K18la) [12, 14]. To overcome the limitations of physiologically limited acyl-CoA concentrations, cells employ a multi-enzyme cooperative system: lysine acyltransferase 2A (KAT2A) and acetyl-CoA synthase 2 (ACSS2) form an acyl-CoA synthase-transferase complex, driving the H3K18 acylation reaction [19]. Beyond histones, non-histone lactylation expands functional repertoires through lactyl-CoA-independent mechanisms. Alanine-tRNA synthetase (AARS1/2), functioning as a lactate-sensing bifunctional enzyme, catalyses lactyl-S-tRNA synthetase-mediated lactylation of key regulatory proteins via an ATP-dependent lactate-adenosine monophosphate intermediate [20]. This includes p53 (acting at sites K120/K139) and cyclic guanosine monophosphate-adenylate cyclase (cGAS acts at site K131) [21, 22]. Complementing these enzymatic pathways, non-enzymatic D-lactylation mediated by the glycolytic derivative S-D-lactylglutathione (SLG) selectively inhibits the NF-κB signalling pathway, establishing a metabolite-driven layer of regulation [23]. The lactylation equilibrium is maintained by deacetylases such as HDAC3, which remove lactylation marks from diverse substrates—including site K673 of the DNA repair protein MRE11—thereby finely regulating cellular processes including DNA repair and gene transcription [24].

**Fig. 1:**
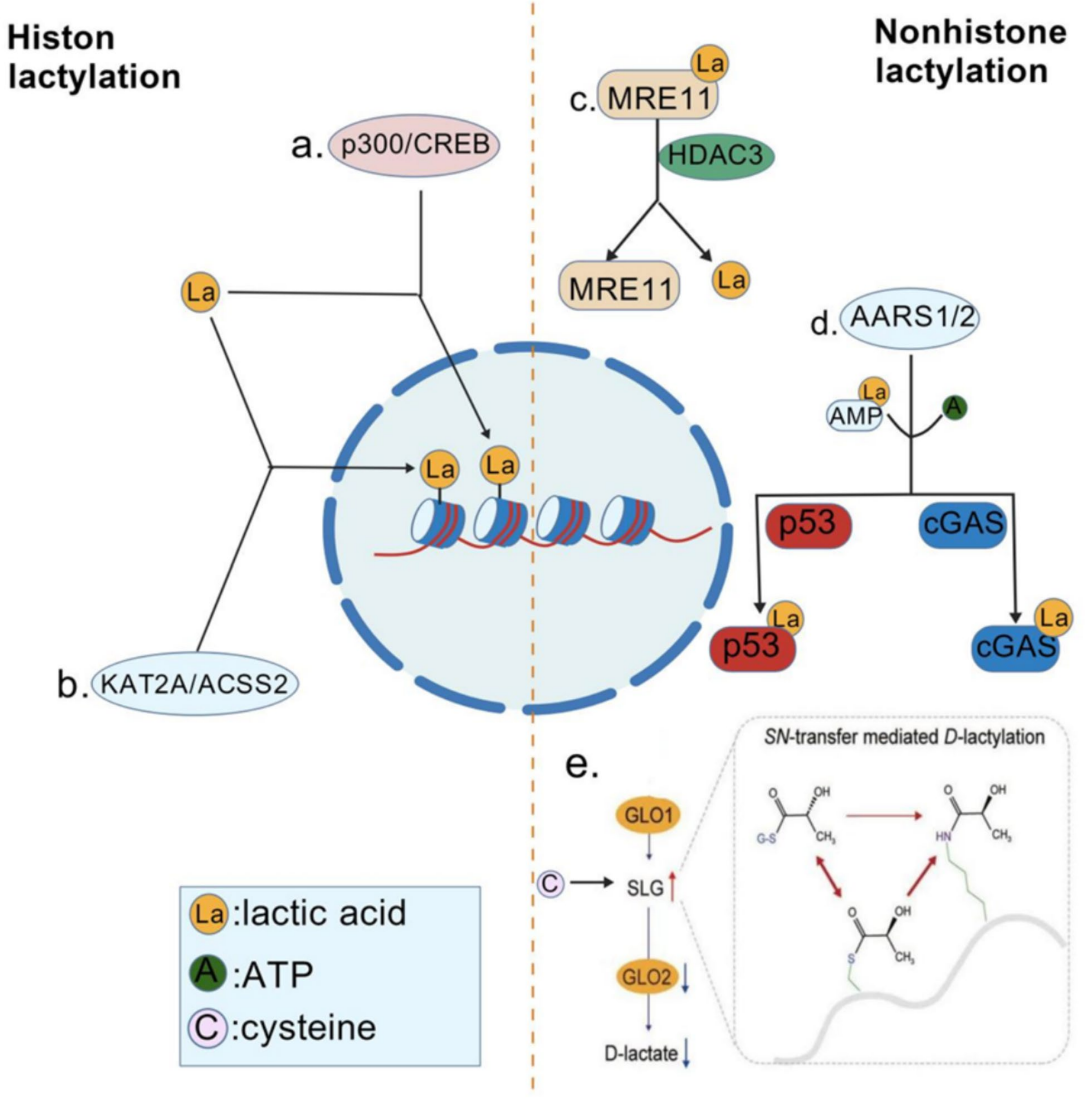
Mechanism of lactylation process. Multiple biochemical pathways and core functions of lactylation. **a** The p300/CREB-binding protein family catalyzes histone H3K18 lactylation dependent on Lactyl-CoA. **b** KAT2A forms a Lactyl-CoA synthetase-transferase complex with ACSS2, driving H3K18 lactylation. **c** HDAC3 inhibits homologous recombination repair activity by removing the lactylation modification on MRE11 at K673. **d** Alanyl-tRNA synthetase AARS1/2 acts as a lactate-sensing bifunctional enzyme, catalyzing lactylation at non-histone sites such as K120/K139 of p53 and K131 of cGAS through an ATP-dependent lactate-adenosine monophosphate intermediate. **e** GLO2 substrate SLG mediates non-enzymatic D-lactylation through neighboring cysteine, selectively inhibiting NF-κB signaling and alleviating inflammation.

The mechanism diagram is shown in Fig. 1.

This intricate modification network exhibits extensive cross-talk with other lysine modifications through shared enzymes, competitive sites, and metabolic regulatory mechanisms. The histone acetyltransferase p300 catalyses both acetylation and lactylation, whilst deacetylases including histone deacetylases (HDACs) remove these modifications, forming dynamic interactions [25, 26]. At shared residue sites such as H3K18, lactylation and acetylation exhibit competitive relationships, exerting opposing effects on fibrotic gene expression [27]. Metabolically, elevated lactate concentrations simultaneously inhibit Sirtuin 1 (SIRT1) activity while enhancing both lactylation and acetylation of high-mobility group box 1 protein (HMGB1), thereby synergistically amplifying inflammatory responses [28]. Furthermore, lactate may function as a signalling molecule directly binding target proteins to induce lactylation via non-enzymatic mechanisms. This allows lactylation to circumvent traditional enzymatic constraints, establishing complementary regulation with classical lysine modifications [28].

The functional significance of this metabolic-epigenetic axis permeates diverse pathological processes. Early studies demonstrated that exogenous lactate stimulates histone lactylation in a concentration-dependent manner [12], while subsequent research has elucidated lactylation’s role in carcinogenesis, DNA repair, and inflammatory regulation. For instance, AARS1-catalysed p53 lactylation impairs its tumour-suppressing function by inhibiting liquid–liquid phase separation and DNA-binding capacity [21, 22]. Concurrently, KAT5-catalysed lactylation at the K388 site of Nijmegen breakage syndrome protein 1 (NBS1) enhances MRN complex assembly and homologous recombination repair efficiency, while HDAC3 dynamically reverses this modification [29]. During inflammation, SLG-induced D-lactylation at the p65 protein K310 site inhibits NF-κB pathway activation [30], demonstrating context-dependent regulatory diversity.

Crucially, this regulatory axis has now been demonstrated to correlate with renal pathology, particularly concerning tubulointerstitial fibrosis. In renal injury models, enhanced glycolysis within tubular epithelial cells leads to elevated lactate levels, significantly increasing lactylated modifications of histone H4K12 [31]. This modification accumulates within the promoter regions of NF-κB pathway genes, promoting their transcription, exacerbating local inflammatory responses, activating fibroblasts, and ultimately driving excessive extracellular matrix deposition [32]. Previous studies have confirmed the role of H3K18 lactylation in driving fibrosis within hepatic and cardiac remodelling models [27, 33], providing a robust mechanistic parallel for understanding renal fibrosis. This positions lactylation as a central regulatory factor in diabetic nephropathy progression and establishes it as a highly promising therapeutic target.

## The role of lactylation modifications in the pathogenesis of DKD

Lactic acid modification has recently been identified as a key epigenetic mechanism linking energy metabolism dysregulation to transcriptional reprogramming in DKD, making it a central focus in DKD pathophysiology research. Beyond its fundamental role as a glycolytic end product, lactic acid also functions as a bioactive signaling molecule. Through non-metabolic regulatory pathways, it directly participates in inflammatory activation and fibrosis progression, playing a pivotal role in the pathogenesis of DKD [34]. Clinical evidence further substantiates lactate's pathological significance: urinary lactate levels in DKD patients show significant positive correlations with renal function indicators such as 24-h urinary albumin excretion and serum creatinine levels [35]. These clinical observations indicate that lactate metabolic imbalance not only serves as a potential biomarker for disease progression but also represents a critical node driving pathological phenotypic alterations. Notably, recent studies reveal marked regulatory heterogeneity in lactate modification across distinct renal cell types—including mesangial cells, podocytes, and tubular epithelial cells—reflecting cell-specific metabolic-epigenetic interactions in DKD progression.

### Lactylation and glomerular injury

Within the glomerular microenvironment under persistent hyperglycaemic conditions, lactylation exhibits cell-type–specific regulatory characteristics, coordinating metabolic reprogramming with epigenetic and signalling events that collectively drive glomerular injury and filtration barrier dysfunction in diabetic nephropathy.

In mesangial cells, high glucose stimulation induces abnormal proliferative activity and promotes excessive extracellular matrix deposition, primarily achieved through upregulating the expression of pro-fibrotic genes such as transforming growth factor-β1, type I collagen, and α-smooth muscle actin. Although direct evidence linking lactylation modification to mesangial cell dysfunction remains limited, emerging research indicates tha elevated lactate levels within mesangial cells under hyperglycaemic conditions induce concurrent lactylation and acetylation of the specific lysine residues (K43/K44) in HMGB1 [36, 37]. This facilitates its transport from the nucleus to the cytoplasm, subsequently activating the NF-κB signalling pathway. This process amplifies inflammatory responses and accelerates fibrotic progression [36]. The hyperglycaemic microenvironment further amplifies oxidative stress via the H4K12 lactylation/NF-κB axis, upregulating Nox4 NADPH oxidase activity and generating substantial ROS in mesangial cells, which not only promotes inflammatory responses but also activates multiple pro-fibrotic pathways including TGF-β, PKC, and AGEs-RAGE [32, 38]*. *Previous evidence indicates that classical epigenetic modifications (particularly histone acetylation and methylation) are definitively involved in the hyperglycaemia-induced mesangial cell fibrosis regulatory network [7], further supporting the potential role of lactylation in mesangial cell regulation. Given the recognised mechanistic similarities between lactylation and acetylation in regulating chromatin accessibility and gene expression patterns [25, 26], it is biologically plausible that lactylation contributes to mesangial cell activation through analogous epigenetic mechanisms. Supporting this inference, histone lactylation has been shown to directly promote extracellular matrix deposition in cardiomyocytes via site-specific chromatin remodelling[39].

In podocytes, the loss of structural and functional integrity is a key determinant of impaired glomerular filtration barrier function [40]. Recent evidence indicates that lactylation significantly promotes podocyte injury through a dual mechanism involving both autophagic dysfunction and phenotypic switching. Under hyperglycaemic conditions, elevated intracellular lactate levels drive both histone lysine lactylation and non-histone lactylation. Elevated histone lactylation promotes podocyte epithelial-mesenchymal transition, manifested by loss of gap junction proteins and acquisition of mesenchymal marker expression. Pharmacological intervention using lactate dehydrogenase inhibitors (such as oxaloacetic acid or dichloroacetic acid) effectively reduces histone lactylation levels, thereby attenuating podocyte transformation and mitigating renal function deterioration [41]. Parallel to histone-mediated effects, non-histone lactylation specifically targets the K970 site of leucyl-tRNA synthetase 1, leading to sustained activation of the mTORC1 signalling pathway. This subsequently inhibits the autophagic flux, ultimately promoting podocyte apoptosis and proteinuria formation in experimental renal disease models [42].

Within glomerular endothelial cells, metabolic epigenetic reprogramming drives microvascular dysfunction [43], with lactylation mediating endothelial inflammatory activation and pro-fibrotic responses through complex epigenetic mechanisms. In-depth investigations reveal that IGFBP5-mediated lactate accumulation induces specific histone H3K18 lactylation, which directly activates the NLRP3 inflammasome complex and promotes IL-1β transcription. This subsequently initiates an endothelium-to-mesenchyme transition characterised by progressive loss of vascular endothelial markers (VE-cadherin, CD31), synchronous acquisition of interstitial markers (α-smooth muscle actin, fibronectin), and excessive extracellular matrix deposition [17, 44]. Concurrently, upregulation of glycolytic enzymes PFKFB3 and PKM induces progressive loss of endothelial markers (VE-cadherin, CD31) alongside synchronous acquisition of stromal markers (α-SMA, fibronectin) and excessive extracellular matrix deposition [17, 44]. Concurrently, the upregulation of glycolytic enzymes PFKFB3 and PKM2 in endothelial cells activates the NF-κB signalling pathway via histone H4K12 lactylation, establishing a pro-inflammatory microenvironment [32]. Notably, although direct evidence within the renal system remains inconclusive, compelling studies in endothelial cells demonstrate that non-histone acetylation modification of the transcription factor TWIST1 at the K150 site enhances its nuclear translocation capacity and directly upregulates TGF-β1 transcription, thereby amplifying fibrotic signalling pathways[45]. Given the significant upregulation of PKM2 during renal glycolytic reprogramming [32], we hypothesise that a similar lactate-mediated regulatory mechanism may operate within glomerular endothelial cells, actively promoting diabetic nephropathy progression through sustained activation of pro-fibrotic pathways.

**Fig. 2:**
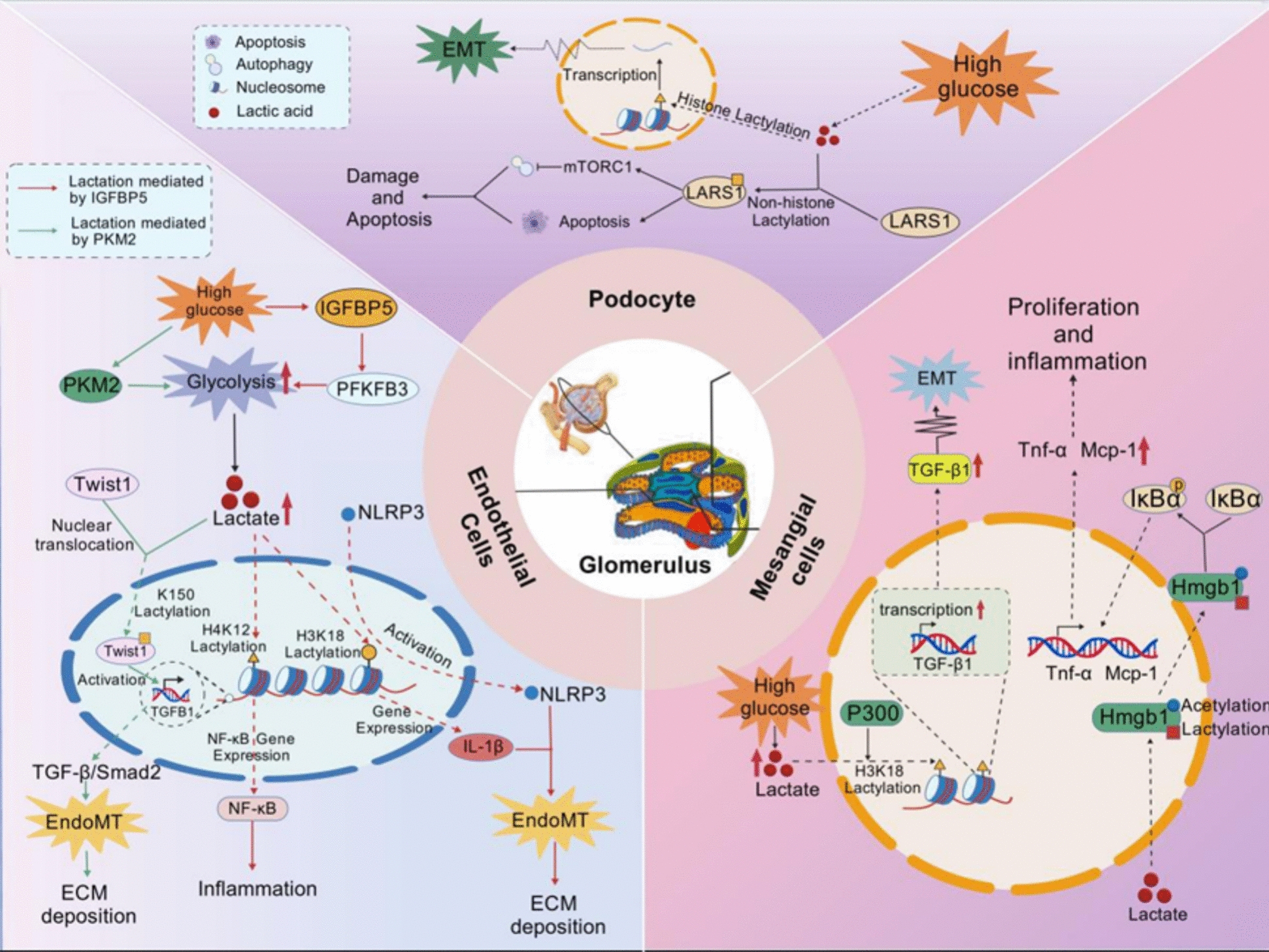
The critical role of lactylation in glomerular injury. Lactylation and glomerular injury involve three components: mesangial cells, podocytes, and glomerular endothelial cells. Mesangial cells exhibit abnormal proliferation and extracellular matrix deposition. While direct evidence for lactylation modifications in mesangial cells is lacking, it is hypothesized that elevated lactate may activate the NF-κB pathway through HMGB1 lactylation/acetylation modifications, contributing to fibrosis. In podocytes, lactylation modifications drive EMT via histone HKla and, through non-histone LARS1-Kla, inhibit autophagy and activate mTORC1 signaling, collectively inducing podocyte apoptosis and protein loss, leading to structural damage and filtration barrier dysfunction. In glomerular endothelial cells, histone H3K18la activates the NLRP3 inflammasome, triggering EndoMT. H4K12la mediates NF-κB inflammatory signaling, while non-histone TWIST1-K150 lactylation may enhance nuclear translocation, upregulating TGF-β1 transcription, thereby promoting microvascular dysfunction and fibrosis.

Figure 2 summarises the mechanism of lactate action in glomerular injury.

### Lactylation and renal tubular injury

Tubulointerstitial injury constitutes a pivotal pathological hallmark of diabetic nephropathy and serves as a strong predictor of long-term renal outcome. This process is closely associated with a fundamental metabolic shift in tubular epithelial cells under hyperglycaemic conditions, characterised by suppression of oxidative phosphorylation and compensatory enhancement of glycolysis, resulting in sustained intracellular lactate accumulation [46]. Rather than functioning solely as a metabolic by-product, lactate acts as a central signalling metabolite that integrates epigenetic reprogramming, mitochondrial dysfunction, cell death pathways, and immune-fibrotic feedback mechanisms to drive progressive tubular injury.

At the level of epigenetic and non-histone reprogramming, recent studies have revealed a finely tuned regulatory network mediated by lactic acid. Lactic acid induces lactylation at histone H3 lysine 14 (H3K14la) in renal tubular cells, forming an open chromatin configuration that promotes transcription of the Krüppel-like factor 5 (KLF5) gene. The upregulated KLF5 protein further binds to specific response elements within the E-cadherin promoter region, effectively suppressing expression of this key epithelial marker while simultaneously promoting transcription of mesenchymal markers including alpha-smooth muscle actin and type I/III collagen. This establishes a molecular framework driving the complete epithelial-mesenchymal transition programme [35]. Beyond histone modifications, our understanding of non-histone lactylation continues to expand. Refined mechanistic studies confirm that lactate synergises with acetylation to jointly modify lysine residues 43 and 44 of HMGB1 protein, disrupting its DNA-binding capacity and liquid–liquid phase separation stability. This dual modification facilitates HMGB1 transport from the nucleus to the cytoplasm, ultimately releasing it into the extracellular space. Here, it functions as a damage-associated molecular pattern molecule, activating the TLR4/NF-κB signalling pathway to amplify the inflammatory response, thereby indirectly yet significantly promoting renal interstitial fibrosis [47].

In the realm of metabolic dysfunction and signal pathway activation, lactate orchestrates intricate metabolic reprogramming processes that progressively exacerbate renal injury. Mass spectrometry analysis reveals lactate directly modifies multiple key mitochondrial enzymes through lactylation, including those within electron transport chain complexes I and III. This disrupts mitochondrial membrane potential and further impairs oxidative phosphorylation capacity. This metabolic disorder exacerbates reactive oxygen species accumulation whilst simultaneously activating the hypoxia-inducible factor-1α and transforming growth factor-β signalling pathways. This establishes a self-perpetuating vicious cycle characterised by ‘glycolysis activation-lactic acid accumulation-mitochondrial dysfunction-metabolic switching’, leading to progressive deterioration of renal tubular injury [48, 49]. Moreover, lactylation specifically targets several rate-limiting enzymes within glycolysis and the pentose phosphate pathway. Proteomic studies indicate that lactylation at position K336 of fructose-6-phosphate kinase 1 enhances its enzymatic activity by approximately 2.1-fold, while lactylation of pyruvate kinase M2 at residues K62 and K305 alters its tetramer formation and allosteric regulation. Collectively, these effects enhance glycolytic flux and induce intermediate metabolite accumulation, thereby reprogramming the energy metabolism of renal tubular epithelial cells towards a pro-fibrotic phenotype [49]. Concurrently, lactylation directly modifies key signalling molecules within the MAPK family (including ERK1/2 and p38) and Akt in the PI3K/Akt pathway. Structural analysis indicates that these lactylation modifications alter protein conformation or accessibility of activation sites, thereby affecting phosphorylation efficiency. This, in turn, regulates key cellular processes such as proliferation, anti-apoptosis, and activation of pro-fibrotic transcriptional programmes via members of the SMAD and SNAIL families [30].

Within the framework of immune-fibrotic feedback mechanisms, emerging evidence reveals how lactate exacerbates renal interstitial fibrosis through complex immunoregulatory actions, thereby establishing a self-sustaining fibrotic microenvironment. Single-cell RNA sequencing analysis of diabetic nephropathy kidney samples indicates that in regulatory T cells, lactate promotes interaction between the membrane-associated spike protein Moxin and TGF-β receptor I by lactylating lysine 72. This significantly amplifies the core fibrotic pathway by enhancing SMAD3 phosphorylation and nuclear translocation, independent of classical TGF-β ligand binding [50]. Concurrently, lactate induces lactylation of pyruvate kinase M2 at position K62 within macrophages, regulating its metabolic reprogramming and promoting polarisation towards a pro-fibrotic M2 phenotype. This phenotype secretes multiple pro-fibrotic factors (including PDGF, CTGF, and IL-13), thereby amplifying fibrotic signalling in adjacent tubular cells and fibroblasts [51]. Furthermore, protein lactylation in T cells critically influences their differentiation fate and functional specialisation. Chromatin immunoprecipitation sequencing analysis confirmed that the lactylation modification of histone H3K18 in CD4 + T cells directly activates FOXP3 gene expression by enriching in its promoter and enhancer regions, thereby promoting regulatory T cell differentiation. Whilst specific lactylation of Ikaros family zinc finger protein 1 at lysine 164 enhances its binding to Th17-associated gene loci (including RORγt and IL-17A) [52], thereby establishing a complex immunometabolic network that sustains chronic inflammation and fibrosis by disrupting the regulatory T cell/Th17 cell equilibrium within the renal microenvironment.

At the level of metabolic dysfunction, lactylation directly targets mitochondrial proteins, thereby linking metabolic reprogramming to organelle injury. Proteomic analyses reveal extensive lactylation sites on mitochondrial enzymes in diabetic renal tissue [53]. Notably, lactylation of acyl-CoA synthetase family member 2 (ACSF2) at lysine 182 inhibits its enzymatic activity, disrupts fatty acid metabolism, promotes reactive oxygen species accumulation, and increases susceptibility to ferroptosis in renal tubular cells [53]. This mitochondrial damage is further exacerbated through lactylation-mediated disruption of the ALDH2/PHB2 axis, leading to impaired mitophagy, cellular energy crisis, and dysregulated cell death execution [54]. Collectively, these events establish mitochondrial dysfunction as an early and central node linking lactylation to tubular injury.

Downstream of mitochondrial damage, lactate-driven histone H4K12 lactylation activates the NF-κB signalling pathway, triggering excessive oxidative stress and inflammation-fibrosis coupling. This pathway upregulates Nox4-dependent NADPH oxidase activity in renal tubular cells, leading to sustained reactive oxygen species generation [33]. Oxidative stress not only induces direct cellular damage but also activates multiple pro-fibrotic signalling cascades, including TGF-β, PKC, and AGEs-RAGE pathways. These processes synergise with NLRP3 inflammasome activation and are further amplified by impairment of the Nrf2 antioxidant defence system, forming a self-reinforcing inflammatory-fibrotic loop [38].

In parallel, lactylation coordinates ferroptosis activation through epigenetic and metabolic convergence. The METTL3-H3Kla axis exerts a bidirectional regulatory effect by upregulating ACSL4 expression while suppressing the GPX4/xCT antioxidant system, thereby disrupting redox homeostasis and sensitising tubular cells to lipid peroxidation-driven ferroptosis [55]. Concurrently, systemic iron dysregulation, including altered transferrin receptor-mediated iron uptake, further accelerates Fenton reactions and ferroptotic injury [56].

Beyond intrinsic renal mechanisms, dysregulation of the gut–kidney axis emerges as an additional contributor to lactate-associated tubular injury. Patients with diabetic nephropathy exhibit reduced short-chain fatty acid production, depletion of lactic acid-producing bacteria, and increased intestinal permeability, facilitating translocation of microbial metabolites into the systemic circulation [57, 58]. Accumulation of these gut-derived metabolites in renal tissue exacerbates oxidative stress and inflammatory injury. Notably, probiotic-based interventions have demonstrated renoprotective effects through restoration of intestinal barrier integrity and enhancement of antioxidant capacity [59].

**Fig. 3:**
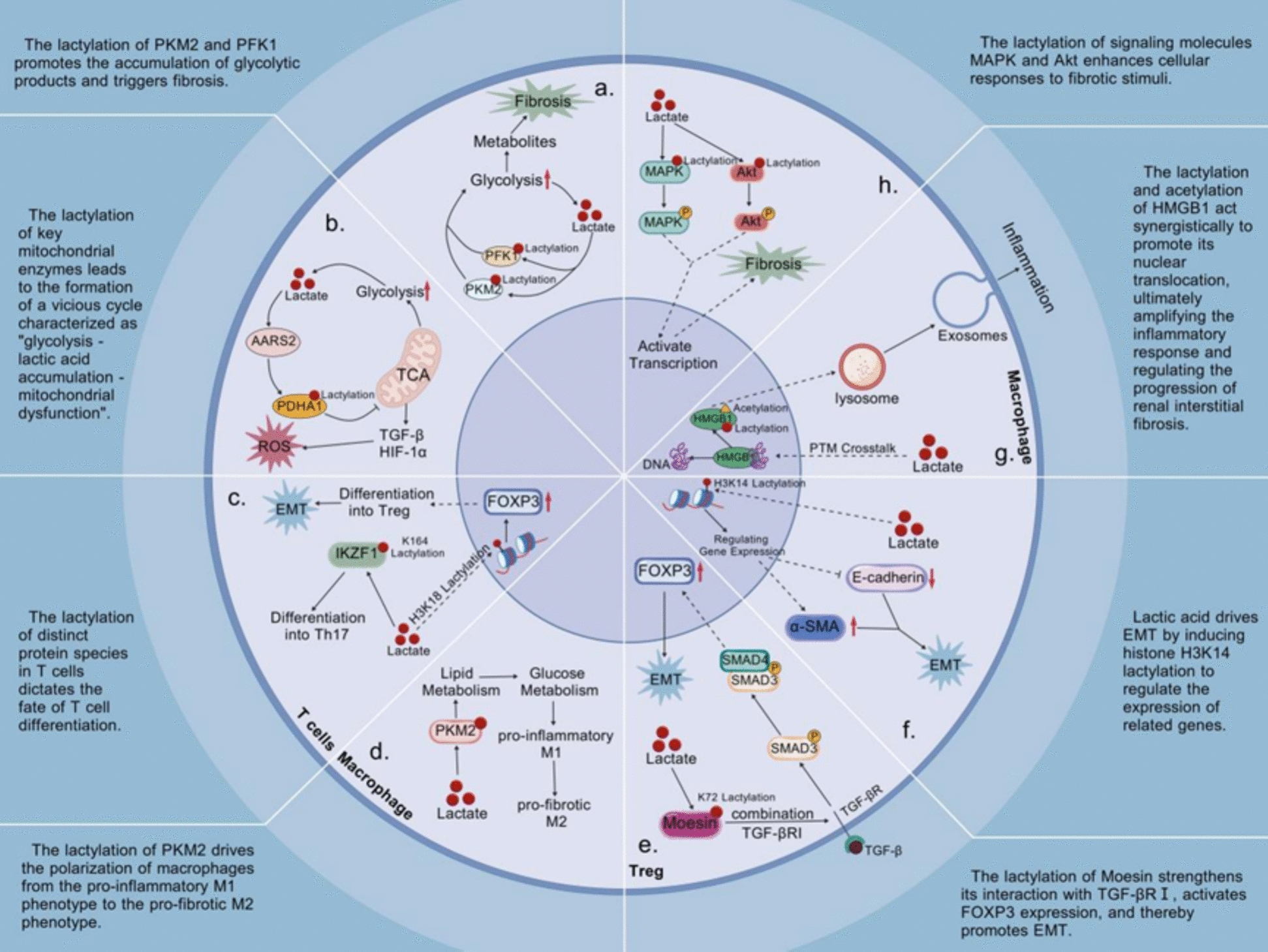
Mechanistic role of lactylation in tubular injury. **a** Lactate-mediated lactylation modifications regulate the enzyme activity of PKM2 and PFK1, thereby enhancing glycolytic flux, promoting metabolite accumulation, and inducing renal tubular epithelial cell fibrosis. **b** Lactate can lactylate key mitochondrial enzymes, inhibiting oxidative phosphorylation and enhancing glycolysis. This cascade reaction promotes ROS accumulation and activates the HIF-1α/TGF-β pathway, forming a vicious cycle of "glycolysis—lactate accumulation—mitochondrial dysfunction." **c** In T cells, lactylation modifications of different proteins determine their differentiation fate: histone H3K18la upregulates the Treg key transcription factor FOXP3, while lactylation at the K164 site of IKZF1 promotes Th17 cell lineage differentiation. **d** Lactylation of PKM2 shifts macrophage metabolism from lipid metabolism to glucose metabolism, promoting polarization from the pro-inflammatory M1 phenotype to the pro-fibrotic M2 phenotype. **e** Lactylation directly modifies signaling molecules such as MAPK and Akt, affecting their phosphorylation efficiency, thereby regulating cell proliferation, anti-apoptosis, and pro-fibrotic transcriptional programs, enhancing the response to fibrosis stimuli. **f** In macrophages, high concentrations of lactate modify crosstalk, disrupting HMGB1's DNA binding and phase separation stability, promoting its release from the nucleus to the extracellular space via the lysosome-exosome pathway, exacerbating inflammation and renal interstitial fibrosis. **g** Lactate coordinates EMT by inducing H3K14 lactylation, activating mesenchymal gene transcription while inhibiting E-cadherin promoter activity, forming a bidirectional regulatory axis that drives the EMT process. **h** In Treg cells, lactate induces lactylation of Moesin at the K72 site, enhancing its interaction with TGF-β1 and synergizing with Smad3 phosphorylation to enhance the TGF-β signaling pathway, thereby activating FOXP3 expression and promoting EMT

The integrated mechanism of lactylation in tubular injury is illustrated in Fig. 3.

## Traditional Chinese medicine in the treatment of diabetic kidney disease: focus on lactylation modifications and extended mechanisms

The traditional Chinese medicine theory of ‘treating disease before it manifests’ provides a unique theoretical framework for understanding diabetic nephropathy. Academician Tong Xiaolin systematically summarised the core pathological mechanism of diabetic nephropathy in traditional Chinese medicine as ‘spleen-kidney deficiency as the root cause, with damp-heat, blood stasis, and toxins as manifestations’, further conceptualising it into three pathological states: deficiency, stasis, and turbidity [53, 54]. This theoretical framework exhibits significant convergence with modern metabolic epigenetics mechanisms. Specifically, the ‘spleen-kidney deficiency’ state corresponds to mitochondrial oxidative phosphorylation dysfunction, triggering compensatory shifts towards glycolysis and resulting in excessive lactate production. Concurrently, manifestations of ‘damp-heat, stasis, and toxins’ foster an acidic microenvironment, inhibit lactate transport, and enhance lactate dehydrogenase activity, thereby establishing a vicious cycle of lactate accumulation and histone lactate modification [16, 38].

Notably, H3K18la serves as a pivotal epigenetic bridge linking metabolic dysfunction to renal fibrosis by activating the transcription of pro-fibrotic genes. This molecular mechanism provides scientific rationale for traditional Chinese medicine's therapeutic strategy of ‘tonifying the spleen and kidneys, unblocking the kidney collaterals, and eliminating turbidity and toxins’. Through this multi-targeted approach, TCM interventions demonstrate unique advantages in regulating the metabolic-epigenetic axis, simultaneously modulating lactate production, lactylation modifications, and downstream fibrotic signalling pathways [35]. Table 1 systematically summarises representative TCM monotherapy and compound formulations targeting this integrated mechanism.

**Table 1:** Summary of Chinese herbal monomers and formulas in regulating lactylation in DKD

Category	Name	Mechanism of action	Key targets/pathways	Experimental/model evidence
Monomers	Baicalin/Baicalein(Flavonoids) [12, 15, 57, 61]	SIRT1/SIRT3 and suppresses p300/CBP-mediated histone lactylation, alleviating pro-inflammatory/fibrotic signaling	HK2,PKM2,LDHA;PI3K/Akt, Raf/MEK/ERK; SIRT1/SIRT3	DKD animal model: reduced proteinuria and renal fibrosis; downregulation of H3K18la and TGF-β1 in renal tissue
	Ginsenosides(Compound K) [58, 59, 62]	Inhibits glycolytic enzyme expression, restores mitochondrial function, removes H3K18la and H4K12la modifications; activates SIRT1, suppresses HDAC4, and reduces ROS	HK2, LDHA; SIRT1/HDAC4; histone H3/H4 lactylation	High-glucose macrophage model: reduced lactate production and decreased ROS; diabetic rat DKD model: improved GFR and alleviated tissue injury
	Cordycepin [63–66]	Activates AMPK–Akt pathway, inhibits PKM2 and LDHA; enhances mitochondrial mitophagy, reduces ROS; indirectly decreases H3K18la, blocking TGF-β/NF-κB signaling	PKM2,LDHA; AMPK-Akt, Mitophagy pathway	Experimental/Model Evidence | High-glucose cell model; DKD animal model: reduced lactate production, alleviated oxidative stress, and improved fibrosis and structural abnormalities
	Evodiamine [67, 89, 90]	Inhibition of LDHA reduces intracellular lactate levels and decreases H3K18 lactylation, thereby suppressing the transcription of pro-fibrotic and pro-inflammatory genes; it also directly inhibits the TGF-β1/Smad signaling pathway, reducing extracellular matrix deposition; moreover, suppression of upstream TGF-β1 diminishes its induction of metabolic reprogramming and lactate production, which, together with direct LDHA inhibition, synergistically enhances the reduction of lactylation modifications	LDHA; TGF-β1/ Smad	Cancer models: directly bind to and inhibit LDHA activity, reducing intracellular lactate levels and H3K18 lactylation; Fibrosis models: inhibit the TGF-β1/Smad signaling pathway
	Salvianolic acid B (Sal B) [60, 70, 71]	Inhibits LDHA activity, reduces lactate accumulation, blocks substrate supply for lactate-dependent inflammatory signaling; inhibits p300/CBP, enhances HDAC3, restores lactylation homeostasis; decreases histone lactylation at promoters of inflammatory and fibrosis-related genes, suppresses pro-inflammatory cytokine release and ROS generation	LDHA;p300/CBP; HDAC3	Liver injury models: Salvianolic acid B regulates lactate production, lactylation-modifying enzymes, and histone lactylation; DKD models: IGFBP5-mediated H3K18 lactylation promotes disease progression
	Triptolide (TP) [72–74]	Downregulates LDHA expression and inhibits HIF-1α stabilisation/nuclear translocation, thereby suppressing glycolytic flux and reducing lactate production. May indirectly attenuate pathological histone lactylation through upstream metabolic inhibition. Exhibits anti-inflammatory and immunosuppressive activities	LDHA; HIF-1α	Tumour models: Markedly suppresses glycolytic flux by downregulating LDHA and inhibiting HIF-1α, reducing lactate production. (Direct evidence linking to lactylation regulation in DKD is currently limited.)
	Aloe-emodin [75–77]	Inhibits P4HB and H3K18 lactylation, alleviates lactylation-mediated ER stress and epigenetic activation, improves cellular metabolism, and suppresses fibrosis gene transcription; inhibits pro-inflammatory pathways such as NF-κB and TGF-β/SMAD fibrotic signaling	Protein disulfide isomerase P4HB (K311la); histone H3K18la; endoplasmic reticulum stress/unfolded protein response (UPR); GOT2-mediated tryptophan metabolism; NF-κB; TGF-β/SMAD	Radiation-induced cardiac injury models: inhibits P4HB lactylation, alleviates fibrosis and functional impairment
	Curcumin [80, 81]	Modulates upstream metabolic signaling pathways (e.g., AKT/mTOR). May indirectly affect the lactate-lactylation axis by influencing glycolysis and inflammatory signaling. Direct regulation of lactylation has not yet been conclusively demonstrated.	Core metabolic pathways (e.g., AKT/mTOR)	Documented ameliorative effects in models of type 2 diabetes and related metabolic disorders
	Berberine(BBR)	Functional inhibition of lactate dehydrogenase A (LDHA) reduces pyruvate-to-lactate conversion; activation of AMPK and suppression of mTORC1 reduces glycolytic reprogramming; restoration of PGC-1α-mediated mitochondrial bioenergetics; suppression of NLRP3 inflammasome and oxidative stress	LDHA (glycolytic enzyme); AMPK/mTORC1 signaling; PGC-1α mitochondrial energy homeostasis; Nrf2 antioxidant pathway	Evidence level: Preclinical; indirect inference from metabolic and signaling regulation in DKD and immune-metabolic models Disease models: Diabetic kidney disease (db/db mice, STZ/HFD rats, HK-2 cell models); immune/inflammatory reprogramming models (e.g., arthritis)
Formula	Yiqi Yangyin Tongluo Formula [82–84]	Inhibition of ROS/JNK signaling, suppression of renal cell apoptosis and COL I deposition; reduction of ROS indirectly decreases lactate production, blocking lactylation-driven EMT and fibrotic signalin	JNK; ROS; Lactate metabolism—histone lactylation—EMT	STZ-induced DKD rats: improved oxidative stress and fibrosis markers
	Shenyi Formula (containing quercetin and kaempferol) [85]	Inhibits aerobic glycolysis; reduces lactate synthesis; synergizes with butyrate to block lactylation-driven fibrotic signaling	HIF-1α/miR-210; Glycolytic pathway	Network pharmacology + animal experiments: decreased lactate production and improvement of DKD phenotypes
	Tangshen Ning Formula, Jisheng Shenqi Pill (Schisandra fruit, Chuanxiong rhizome, Oyster shell) [86]	Upregulation of Nrf2/HO-1/GPX4; suppression of TNF-α and MCP-1; activation of the TFAM pathway; improvement of mitochondrial oxidative phosphorylation and reduction of lactate production, indirectly inhibiting lactylation-driven activation of fibrotic genes such as TGF-β1 and α-SMA	Nrf2; TFAM; ROS; Mitochondrial function; Lactate metabolism	Clinical/animal evidence: reduced proteinuria; improvement in oxidative stress, inflammation, and fibrosis markers; presumed involvement of lactylation-related regulation
	Danggui Buxue Decoction/Danggui Shaoyao San [87, 88]	Downregulation of TGF-β1/Smad3, COL IV, and α-SMA; suppression of IL-6, TNF-α, and CRP; inhibition of ECM deposition; potentially reducing lactylation-driven profibrotic transcriptional activity	TGF-β/Smad; Pro-inflammatory factors; Lactylation pathway	GK rats, STZ-induced DKD models: improved fibrosis/inflammation markers; presumed modulation of lactylation-related epigenetics

### Regulatory effects of traditional Chinese medicine monomers on lactate metabolism and lactylation

Beyond elucidating the metabolic epigenomic pathomechanisms of DKD, research has progressively revealed how specific single-herb Chinese medicinal preparations precisely target the lactate-lactylation axis. In diabetic nephropathy, abnormally activated glycolysis and the resulting lactic acid accumulation not only disrupt cellular energy metabolism but also regulate inflammatory and fibrotic signalling networks through histone lactylation modifications, thereby promoting glomerulosclerosis and tubulointerstitial fibrosis [16, 32]. Moreover, lactate is now recognised as a key signalling molecule and endogenous substrate for histone lysine lactylation, regulating metabolic-epigenetic interactions through multiple enzymatic and non-enzymatic pathways [35, 55, 56]. Within this pathological framework, Chinese herbal monomers (including baicalin, ginsenosides, cordycepin, evodiamine, tannic acid B, curculigin A, and aloe-emodin) have demonstrated significant efficacy in regulating lactate metabolism and lactylation through multi-target mechanisms [60]. Their intervention primarily operates through three strategies: firstly, inhibiting key glycolytic enzymes such as PFKFB3 and LDHA to reduce lactate production at its source; secondly, modulating lactate transporters including monocarboxylate transporters to enhance lactate clearance; and finally, directly influencing the activity of lactate-related regulatory proteins to correct pathological hyperlactacidosis. These synergistic effects effectively disrupt the self-amplifying metabolic signalling network driven by lactic acid imbalance, thereby mitigating structural damage to the kidneys [60].

As representative flavonoid constituents, baicalin and baicalein have been widely investigated for their renoprotective effects in DKD, particularly with respect to metabolic dysregulation and renal fibrosis [57]. Under hyperglycaemic conditions, renal cells exhibit enhanced glycolytic activity, characterized by the upregulation of key enzymes such as PFKFB3, HK2, PKM2, and LDHA, leading to lactate accumulation and impaired mitochondrial oxidative phosphorylation [16]. Emerging evidence indicates that lactate may function as a signalling metabolite capable of modulating inflammatory and fibrotic gene expression through histone lactylation, thereby contributing to DKD progression [12, 32, 34]. At present, direct experimental evidence linking baicalin to the regulation of histone lactylation in DKD is unavailable. Nevertheless, existing studies suggest a potential indirect association. Baicalin has been reported to suppress glycolytic enzyme expression via inhibition of the PI3K/Akt and Raf/MEK/ERK pathways in inflammatory and metabolic disease models [15], which may reduce intracellular lactate production. In addition, baicalin can enhance the activity of deacetylases such as SIRT1 and SIRT3 in diabetic cardiomyopathy and related metabolic disorders [61], indicating a possible influence on epigenetic regulatory processes. In DKD animal models, baicalin administration significantly attenuates renal inflammation and fibrosis, as evidenced by decreased expression of TGF-β1, IL-1β, and Col1a1, along with improved histopathological features [57]. Collectively, these findings support the therapeutic potential of baicalin in DKD and raise the possibility that its metabolic regulatory effects may intersect with lactate-associated epigenetic mechanisms, although direct validation of this hypothesis in renal tissue remains to be established.

Ginsenosides, as the primary bioactive saponins among ginseng metabolites, demonstrate considerable potential in modulating metabolic reprogramming associated with diabetic nephropathy. Among these, compound K significantly downregulates key glycolytic enzymes such as HK2 and LDHA, reducing lactate production while restoring mitochondrial oxidative phosphorylation capacity. Although its precise renal functions require direct validation, studies in high-glucose-stimulated macrophages and inflammatory models indicate that compound K effectively corrects abnormal glycolytic gene expression and energy metabolism disorders through a dual regulatory mechanism—activating SIRT1 while inhibiting HDAC4—significantly reducing reactive oxygen species levels [58]. Moreover, compound K promotes the reversal of histone acetylation modifications via an HDAC/SIRT-mediated deacetylation mechanism. By facilitating the removal of acetylation marks at specific histone sites such as H3K18 and H4K12, compound K subsequently inhibits the activation of downstream NF-κB and TGF-β signalling pathways, ultimately mitigating mitochondrial damage and oxidative stress within the renal tubular environment [59]. In diabetic animal models treated with compound K, renal tissue analysis revealed restored glomerular filtration rate, reduced urinary protein excretion, and significant improvement in characteristic histopathological alterations including thickened glomerular capillary basement membranes and mesangial expansion [62].

Cordycepin, as a well-studied active small molecule in traditional Chinese medicine, primarily exerts its renal protective effects by regulating glycolytic metabolism and lactate modification through modulation of the AMPK-Akt signalling axis. Experimental evidence indicates that Cordycepin directly activates AMPK signalling whilst simultaneously modulating Akt activity, leading to downregulation of key glycolytic enzymes PKM2 and LDHA. This synergistic regulation effectively reduces lactate production, alleviating associated extracellular acidosis and metabolic stress [63–65]. Following AMPK activation, cordycepin further promotes mitochondrial autophagy and functional recovery, accelerating the clearance of damaged mitochondria and reducing reactive oxygen species accumulation. The restoration of cellular energy homeostasis subsequently reverses hyperglycaemia-induced glucotoxicity and ferroptosis [64, 65]. Given that lactate is an essential substrate for lactate modification, cordycepin's reduction of lactate levels indirectly diminishes H3K18la accumulation. Concurrently, AMPK-activated repair mechanisms may directly promote normalisation of lactate modification, thereby blocking lactate-driven epigenetic activation signals. This dual action attenuates activation of TGF-β and NF-κB pathways, ultimately suppressing expression of inflammatory cytokines and chemokines [65, 66].

Evodiamine, the primary alkaloid component of the traditional Chinese medicinal herb Evodia, demonstrates significant potential in regulating the metabolic microenvironment associated with diabetic nephropathy. Tumour biology research confirms that evodiamine exerts potent inhibitory effects by directly binding to the catalytic site of lactate LDHA, substantially reducing intracellular lactate concentrations and thereby diminishing the availability of substrates required for histone lactylation modification [67]. In these models, evodiamine‑mediated reduction in lactate‑linked histone lactylation suggests its ability to intersect metabolic and epigenetic regulation in pathological states driven by enhanced glycolysis and lactate accumulation [67]. Elevated lactate and associated histone lactylation have been implicated in DKD progression by promoting accelerated tubular epithelial cell senescence and fibrotic responses; downregulation of histone lactylation by Glis1 alleviates renal tubular cell senescence and reduces fibrosis in DKD, linking metabolic reprogramming to epigenetic control of cellular aging and fibrogenic pathways in the diabetic kidney [68]. Evodiamine’s modulation of lactate metabolism and histone lactylation is complemented by its systemic protective effects relevant to DKD. In diabetic and renal injury models, evodiamine improves hyperglycemia, insulin resistance, oxidative stress, and inflammation, while ameliorating renal pathological changes [69]. Taken together, these findings suggest that evodiamine may simultaneously target lactate‑driven epigenetic dysregulation and systemic metabolic‑inflammatory disturbances, highlighting its potential as a multifaceted therapeutic agent to mitigate tubular injury and fibrotic progression in diabetic kidney disease.

Sal B, as the primary water-soluble bioactive compound extracted from Salvia miltiorrhiza, demonstrates potential renoprotective effects in diabetic nephropathy through multi-level regulation of lactate-related pathways. Although evidence linking Sal B directly to lactate modification in diabetic nephropathy warrants further investigation, studies in liver injury models provide compelling mechanistic insights. Research indicates that Sal B effectively suppresses lactate production by inhibiting the glycolytic flux driven by lactate dehydrogenase A in macrophages, thereby reducing the substrate supply for lactylation modification [70]. Moreover, Sal B exhibits bidirectional regulation of lactyl-modifying enzymes: it inhibits p300/CBP lactyl transferase activity while enhancing HDAC3's de-lactylation function, thereby synergistically restoring dynamic equilibrium in histone lactylation [60]. At the transcriptional level, Sal B treatment reduces the enrichment of histone lactylation in the promoter regions of pro-inflammatory and pro-fibrotic genes, thereby inhibiting the release of inflammatory cytokines and decreasing reactive oxygen species production [71].Given the established mechanistic parallels between IGFBP5-mediated H3K18 lactylation-driven endothelial-to-myocyte transition (EndoMT) in renal fibrosis and analogous processes in hepatic injury models [44], and considering Sal B's demonstrated multi-target regulation of the lactylation axis, this compound warrants investigation as a promising therapeutic candidate to correct dysregulated lactylation in the pathophysiology of DKD.

Triptolide, a diterpene epoxide isolated from Tripterygium wilfordii Hook. f., exhibits potent anti-inflammatory and immunosuppressive activities and has been increasingly implicated in the regulation of metabolic reprogramming, including modulation of immune cell–associated inflammatory responses [72]. Although direct evidence linking triptolide to lactylation regulation in diabetic nephropathy is currently limited, tumour model studies demonstrate that triptolide markedly suppresses glycolytic flux by downregulating LDHA expression and inhibiting hypoxia-inducible factor-1α (HIF-1α) stabilisation and nuclear translocation, thereby reducing lactate production [73, 74]. Given the central role of lactate accumulation in driving histone lactylation and pro-fibrotic gene transcription in diabetic kidney disease [34, 35, 40, 46], these findings suggest that triptolide may indirectly attenuate pathological lactylation through upstream metabolic inhibition.

Aloe emodin is a natural anthraquinone compound derived from medicinal plants including the genera Aloe and Rheum, exhibiting multiple biological activities encompassing anti-inflammatory, antioxidant, and immunomodulatory properties. Increasing evidence indicates this compound demonstrates significant efficacy in improving energy metabolism, inhibiting apoptotic pathways, and alleviating tissue fibrosis in various metabolic disease models [75, 76]. Recent mechanistic studies reveal that aloe-emodin concurrently regulates glycolytic pathways, mitochondrial function, and protein lactylation modifications, thereby exerting synergistic effects on cellular metabolism and epigenetic networks [77]. In radiation-induced cardiac injury models, aloe emodin was found to specifically inhibit lysine 311 lactylation of protein disulfide isomerase P4HB, thereby alleviating endoplasmic reticulum stress and the unfolded protein response triggered by altered P4HB structure and function. Concurrently, this compound rectified GOT2-mediated tryptophan/kynurenine metabolic imbalance, ultimately mitigating cardiac fibrosis and dysfunction. These therapeutic benefits correlate with reduced levels of the histone lactylated mark H3K18la [77].

Although this specific mechanism remains to be directly validated in diabetic kidney disease, it is reasonable to hypothesise that aloe-emodin may exert similar effects given the shared pathological features between diabetic kidney disease and radiation-induced tissue injury—including enhanced glycolytic flux, lactic acid accumulation, endoplasmic reticulum stress, mitochondrial dysfunction, and activation of the fibrosis transcriptional programme [14, 16, 17]. Consequently, aloe-emodin may ameliorate DKD progression via a dual mechanism: by inhibiting lactate production whilst simultaneously blocking lactylated modifications of key proteins (particularly through regulating H3K18la levels), thereby disrupting the self-perpetuating cycle of ‘metabolic disorder-lactylation-epigenetic activation’ within renal tissue. Beyond these primary mechanisms, studies confirm aloe-emodin also inhibits the NF-κB inflammatory signalling pathway and TGF-β/Smad pro-fibrotic pathways [78, 79]. These synergistic actions hold promise for improving the immunometabolic microenvironment, further substantiating its multi-target therapeutic potential for diabetic kidney disease.

In addition to aloe emodin, accumulating evidence indicates that lactate accumulation and lactate-driven immune–metabolic reprogramming play a critical role in shaping inflammatory responses in metabolic diseases, including diabetic kidney disease. In particular, suppression of NF-κB activation and pro-inflammatory cytokine release in macrophages has been shown to attenuate immune–metabolic reprogramming associated with lactate accumulation [12]. Within this mechanistic framework, certain natural compounds have been reported to modulate upstream metabolic signalling pathways. For example, curcumin has been documented for its ameliorative effects on type 2 diabetes mellitus and related metabolic disorders [80], which may encompass the regulation of central pathways such as AKT/mTOR that govern glycolytic activation and cellular metabolic reprogramming [81]. Although direct regulation of protein or histone lactylation by these compounds has not yet been conclusively demonstrated in diabetic kidney disease models, their established effects on glycolysis, lactate accumulation, and inflammatory signalling provide supportive evidence that multiple traditional Chinese medicine monomers may converge on the lactate–lactylation axis through complementary upstream mechanisms.

TCM monomers collectively establish a multi-tiered therapeutic strategy, concurrently achieving lactate reduction, lactacidification regulation, energy metabolism restoration, mitochondrial repair, and suppression of pro-inflammatory and pro-fibrotic signalling pathways. This integrated approach functions through multiple synergistic mechanisms: firstly, by inhibiting key glycolytic enzymes and modulating lactate transport systems, TCM monomers effectively diminish lactate production and efflux, thereby preventing the establishment of an acidic microenvironment and subsequent lactate-driven epigenetic activation; Secondly, by activating the SIRT/HDAC system and modulating the AMPK/Akt signalling axis, these compounds promote the clearance of lactate-mediated modifications, restoring normal transcription of inflammatory and fibrotic genes; Thirdly, by restoring metabolic homeostasis through pathways such as AKT/GSK3β and AMPK-mTOR whilst enhancing mitochondrial autophagy and biogenesis, TCM monomers achieve comprehensive intervention spanning fundamental energy metabolism to cellular signalling and gene expression networks. This enables precise targeting of multiple critical nodes within the ‘metabolic-inflammatory-fibrotic’ axis in diabetic nephropathy.

A growing body of experimental evidence corroborates this therapeutic approach. In diabetic nephropathy models, baicalin treatment significantly improved the albumin-to-creatinine ratio, glomerular basement membrane thickness, and mesangial expansion [57]. K compound intervention markedly enhanced renal reserve capacity and reduced fibrosis biomarker levels [62]. Cordycepin demonstrated significant efficacy in alleviating oxidative stress, suppressing inflammation, and repairing renal structural abnormalities [66]. Although the lactate-modulating effects of certain monomeric compounds require further direct validation within DKD-specific environments, existing mechanistic evidence provides robust justification for their application in DKD treatment. Collectively, these findings align with a reconceptualised pathological framework centred on lactate metabolism: TCM monomers exert their effects through sequential interventions—reducing lactate production to block initiation signals, clearing lactate modifications to correct transcriptional dysregulation, restoring energy homeostasis to maintain cellular integrity, and suppressing pro-inflammatory and pro-fibrotic pathways to improve the structural and functional pathology of diabetic nephropathy.

### Systemic regulation of diabetic kidney disease by traditional Chinese medicine formulas

Building upon the established framework—where specific Chinese herbal monomers demonstrate significant efficacy in alleviating renal inflammation and fibrosis by modulating lactate modification—traditional Chinese medicinal formulas present complementary therapeutic strategies. These complex formulations, by integrating multiple bioactive components with synergistic effects, hold promise for implementing more comprehensive and systematic interventions within the lactate regulation network. The inherent multi-component nature of TCM formulations enables simultaneous regulation of lactate production, transport, and utilisation pathways, while concurrently targeting enzymatic mechanisms governing lactacidification dynamics and their downstream pathological consequences. This systems-level intervention strategy aligns with the multifactorial pathogenesis of diabetic nephropathy. By implementing synergistic mechanisms at multiple nodes along the lactacidification axis, it holds promise for achieving optimised therapeutic outcomes.

The Qi-tonifying, Yin-nourishing and Meridian-unblocking Formula is a classical Chinese medicine prescription developed for the intervention of diabetic nephropathy. Its therapeutic rationale is grounded in four principles: tonifying qi, nourishing yin, invigorating blood circulation and resolving stasis, and unblocking meridians. Experimental studies utilising streptozotocin-induced diabetic nephropathy rat models demonstrated that this formula significantly reduces renal reactive oxygen species levels, downregulates phosphorylated c-Jun N-terminal kinase expression, and concurrently inhibits renal cell apoptosis and abnormal type I collagen deposition [82]. The ROS/JNK signalling pathway, as a key mediator of cellular oxidative stress and epithelial-mesenchymal transition (EMT), represents a crucial mechanism through which this formula indirectly inhibits EMT progression and secondary renal interstitial fibrosis via a cascade reaction involving hyperglycaemia-induced metabolic lactate accumulation and histone lactoylation modification [83]. Furthermore, the formula-mediated reduction in oxidative stress contributes to improved energy metabolic homeostasis, thereby decreasing lactate production. This subsequently weakens the epigenetic regulation of fibrotic signalling through histone lactoylation modification while supporting the restoration of mitochondrial oxidative phosphorylation function [84].

The Shenyi Formula (SY Formula), containing bioactive components such as quercetin and kaempferol, has been demonstrated through comprehensive network pharmacology analysis to possess multi-target regulatory properties. These studies reveal extensive associations between the formula and key signalling pathways, including the receptor for advanced glycation end-products (RAGE), phosphoinositide 3-kinase-protein kinase B (PI3K-Akt), and interleukin-17 (IL-17) cascades. They demonstrate therapeutic efficacy in suppressing abnormal TGF-β expression within glomerular structures [85]. Experimental validation further indicates that the core component quercetin specifically targets the HIF-1α/miR-210 regulatory axis, effectively inhibiting aerobic glycolysis and thereby significantly reducing lactate biosynthesis [85]. This metabolic reprogramming mechanism resonates with sodium butyrate's pharmacological activity in inhibiting lactic acid modification through cellular metabolic regulation. Collectively, these findings confirm that the SY formulation alleviates renal interstitial fibrosis driven by pathological lactic acidification by reducing the burden of lactate production.

Clinical and experimental studies have confirmed that several proprietary Chinese medicinal formulations—including Tang Shen Ning, Ji Sheng Bu Shen Wan, and modified formulations containing schisandra, chuanxiong, and oyster shell—can significantly reduce pathological proteinuria in diabetic nephropathy patients while alleviating oxidative stress and inflammatory responses. This effect is achieved under the therapeutic principle of ‘tonifying the kidneys and nourishing essence, promoting blood circulation and resolving stasis’. These beneficial effects are primarily mediated through upregulation of the Nrf2/HO-1/GPX4 pathway, thereby reducing the expression of pro-inflammatory factors such as TNF-α and MCP-1 while improving renal microcirculation and mitochondrial function. Enhanced mitochondrial integrity is achieved through dual activation of the Nrf2 and TFAM pathways, thereby improving mitochondrial bioenergetics and optimising autophagy regulation. These synergistic effects collectively contribute to its multidimensional renal protective effects [86]. Based on the established mechanisms above, it is reasonable to hypothesise that this formulation may restore mitochondrial oxidative phosphorylation capacity by repairing microcirculation and improving oxygen supply. This, in turn, reduces lactate production, thereby further delaying the progression of diabetic nephropathy. This metabolic normalisation would subsequently attenuate lactate-dependent histone lactylation and its transcriptional activation of pro-fibrotic genes such as TGF-β1 and α-SMA, ultimately diminishing renal interstitial fibrosis signalling pathways [34, 35]. Although this mechanistic hypothesis aligns strongly with established links between metabolic reprogramming and epigenetic regulation in diabetic nephropathy, direct experimental validation remains necessary. This should involve comprehensive assessment of renal lactate levels, specific lactate modification markers, and fibrosis-related gene expression profiles to confirm this epigenetic pathway as a contributing mechanism to the therapeutic efficacy of these formulations.

Danggui Buxue Tang and Danggui Shaoyao San have been extensively validated in diabetic nephropathy models, exhibiting dual anti-inflammatory and anti-fibrotic properties. Experimental studies indicate that following eight weeks of continuous administration in GK diabetic rat models, Danggui Buxue Tang significantly downregulated TGF-β1 and Smad3 expression whilst simultaneously upregulating inhibitory Smad5 activity. It further reduced protein levels of type IV collagen and α-smooth muscle actin, ultimately mitigating pathological extracellular matrix deposition within renal interstitial tissue [87]. Furthermore, this formula effectively reduced expression of pro-inflammatory mediators (including IL-6, TNF-α, and C-reactive protein), thereby mitigating oxidative stress and inflammation-related renal injury [87]. Supplementary studies on Angelica and Peony Powder in streptozotocin-induced diabetic models revealed analogous mechanisms: significant inhibition of the TGF-β/Smad pathway, reduced fibronectin and type IV collagen deposition, and downregulation of NF-κB activity. Collectively, these findings demonstrate its capacity to concurrently suppress inflammatory responses and extracellular matrix accumulation [88]. Based on these established mechanisms of action, and in light of recent evidence suggesting that TGF-β pathway inhibition may attenuate histone H3K14la lactylation [35], it is reasonable to hypothesise that these formulations may additionally modulate the epigenetic dimension of renal fibrosis by reducing lactylation-driven transcriptional activation of pro-fibrotic genes. This potential mechanism warrants further investigation to comprehensively elucidate the relationship between traditional formula-mediated TGF-β inhibition and contemporary understanding of metabolic-epigenetic interactions in diabetic nephropathy.

Traditional Chinese medicine demonstrates multi-tiered regulatory mechanisms in intervening against the pathological progression of diabetic nephropathy. Its action pathways may encompass a continuum of biological pathways, ranging from inhibiting glycolysis to reducing lactate production, ultimately regulating lactate modification. Single-herb TCM formulations primarily regulate glucose metabolic reprogramming by directly targeting key enzymes or signalling molecules, thereby influencing lactate production and subsequent lactylation modifications. Conversely, TCM compound formulations leverage their inherent multi-component, multi-target advantages to systematically modulate metabolic-epigenetic crosstalk, promoting comprehensive remodelling of the renal microenvironment [57, 82, 85, 86].

From the perspective of traditional Chinese medicine theory, the pathological triad of ‘deficiency-stasis-turbidity’ conceptually corresponds to mitochondrial dysfunction, lactic acid metabolism disorders, and abnormal histone modifications within the modern pathological cascade [53, 54]. Adhering to the ‘state-target synchronisation’ therapeutic principle, TCM interventions enhance energy metabolism by fortifying the spleen and tonifying the kidneys, unblock the kidney meridian to interrupt fibrosis signalling pathways, and transform turbidity and eliminate toxins to regulate the acidic microenvironment [16, 35]. This integrated therapeutic strategy comprehensively addresses lactate modification and its downstream fibrotic progression. It not only offers novel perspectives for targeting lactate-mediated epigenetic interventions but also highlights traditional Chinese medicine's unique advantages in integrating metabolic regulation with epigenetic modifications.

Taken together, these findings highlight the potential intersection between TCM-mediated metabolic regulation and lactylation-associated epigenetic mechanisms in DKD. However, it should be noted that, for most compounds discussed, the proposed involvement of lactylation is primarily inferred from upstream metabolic and signaling alterations rather than from direct, site-specific lactylation measurements in renal tissue. These conceptual and methodological constraints are further discussed in the concluding section.

### Clinical perspectives on targeting lactylation in diabetic kidney disease

The recognition of lactylation represents a pivotal shift in the clinical conceptualization of DKD, moving beyond conventional hemodynamic and hormonal paradigms to address a core metabolic–epigenetic driver of progression. This axis directly links the pathological glycolytic shift observed in diabetic kidneys to the sustained activation of pro-fibrotic and pro-inflammatory gene programs [35, 91, 92]. Consequently, its clinical relevance is profound: it provides a mechanistic explanation for the heterogeneous treatment response observed in practice and introduces a measurable pathological process—dysregulated lactate metabolism—that can be therapeutically targeted [93]. This reframes the clinical challenge from merely slowing decline to potentially modifying a fundamental disease pathway.

From a practical standpoint, this axis offers a tangible tool for patient stratification and prognostic refinement. Elevated systemic and urinary lactate levels, corroborated by upregulation of glycolytic enzymes in renal tissue, may delineate a “high-lactate” DKD phenotype characterized by more aggressive fibro-inflammatory activity and a potentially faster trajectory of renal function loss [91]. Monitoring lactate dynamics could therefore transition from a research observation to a clinical strategy for identifying high-risk patients who might benefit from intensified or targeted interventions, enabling a move towards precision management in a field historically dominated by a one-size-fits-all approach.

TCM is uniquely positioned to intervene within this new framework. Its foundational principles of “clearing heat, resolving dampness, and invigorating blood circulation” offer a striking conceptual parallel to counteracting the lactate-accumulated, pro-inflammatory, and pro-fibrotic milieu central to DKD pathology [86]. Critically, numerous TCM compounds and formulas with documented clinical benefits in mitigating DKD symptoms—such as the Shenyi Formula, berberine, or Danggui Buxue Decoction—exert multifaceted effects that converge on this very axis. Their reported capacities to modulate cellular metabolic reprogramming, enhancing mitochondrial function, and attenuating downstream inflammatory and fibrotic signaling provide a robust and coherent scientific rationale for their efficacy [86, 94]. Thus, TCM emerges not as an adjunct but as a rational, system-based therapeutic strategy capable of restoring balance to the dysregulated metabolic–epigenetic network at the heart of DKD.

Translating this integrated perspective into clinical practice necessitates a deliberate research pathway. Future clinical trials, especially those evaluating TCM interventions, should prioritize the incorporation of lactate-related biomarkers (e.g., urinary lactate-to-creatinine ratio) as exploratory endpoints to directly test the hypothesis that modulating this axis underpins therapeutic benefit [91, 93]. Furthermore, investigating the synergistic potential of combining standardized TCM formulations with foundational therapies like SGLT2 inhibitors is crucial. Such combination studies will assess whether concurrent targeting of lactylation and other pathways yields additive or synergistic renal protection, particularly for the identified high-risk phenotypes [93, 95]. This approach embodies the integration of TCM’s holistic, syndrome-differentiation principles with modern biomarker-driven stratification.

In conclusion, incorporating lactylation into the clinical paradigm of DKD opens a transformative path for integrated management. It enriches our understanding of disease heterogeneity, provides a mechanistic scaffold for the therapeutic action of TCM, and charts a clear course for future clinical validation.

## Conclusion and future perspectives

Accumulating studies indicate that lactylation represents an important metabolic–epigenetic mechanism contributing to fibrotic progression in diabetic nephropathy through cell-type-specific pathways. In tubular epithelial cells, hyperglycaemia-driven glycolytic activation has been shown to induce H3K14 lactylation–dependent activation of Krüppel-like factor 5, thereby promoting epithelial–mesenchymal transition [35]. In glomerular endothelial cells, IGFBP5-mediated H3K18 lactylation activates the NLRP3 inflammasome and facilitates endothelial–mesenchymal transition [44], while in podocytes, lactylation-associated modulation of the PI3K/AKT/NF-κB pathway disrupts the expression of key slit diaphragm and cytoskeletal proteins such as CD2AP and WT-1, ultimately impairing the integrity of the glomerular filtration barrier [38]. Collectively, these findings outline a multidimensional pathogenic network linking metabolic dysregulation, epigenetic reprogramming, and inflammatory activation in diabetic nephropathy.

Within this framework, TCM exhibits distinct therapeutic characteristics through its multi-target and multi-level regulatory effects. Single-herb interventions have been reported to modulate key metabolic enzymes and signalling pathways involved in lactate production and lactacidification [67, 91], whereas classical compound formulations exert synergistic actions that contribute to the holistic remodelling of the renal microenvironment [96, 97]. Notably, these pharmacological features are conceptually consistent with the traditional Chinese medicine theory of “deficiency–stasis–turbidity”, which shows notable parallels with contemporary understandings of mitochondrial dysfunction, disordered lactate metabolism, and aberrant histone modifications in diabetic nephropathy [62, 63].

Despite these advances, several important limitations should be acknowledged. First, although cell-type-specific roles of lactylation have been described [35, 38, 44], most evidence is derived from experimental models, and direct validation in human renal tissue remains limited. Second, the regulatory effects of TCM on lactylation are predominantly inferred from upstream metabolic or signalling changes rather than from direct assessment of site-specific lactylation modifications, which constrains causal interpretation. Third, many studies rely on bulk tissue analyses, potentially obscuring the spatial and cellular heterogeneity of lactylation dynamics within the kidney. In addition, the lack of standardized detection methods and quantitative criteria for lactylation hampers cross-study comparison and reproducibility.

From the perspective of TCM research, the intrinsic complexity of multi-herb formulations further complicates mechanistic dissection, making it challenging to attribute specific epigenetic outcomes to individual components or defined combinations. Moreover, the evidence summarized in this review is largely preclinical, and systematic clinical investigations integrating lactylation-related biomarkers with TCM syndrome differentiation are still scarce.

Future research should therefore focus on integrating single-cell and spatial multi-omics approaches to resolve cell-specific lactylation patterns, alongside the development of standardized and quantitative methodologies for lactylation detection. Importantly, mechanistic studies directly examining how TCM interventions influence lactylation “writers”, “erasers”, and site-specific modifications in renal tissue are warranted. Addressing these challenges will be essential for translating lactylation-targeted strategies into more precise and evidence-based applications of traditional Chinese medicine for diabetic nephropathy.

## Data Availability

No datasets were generated or analysed during the current study.

## Abbreviations

DKD: Diabetic kidney disease
LDHA: Lactate dehydrogenase A
LDHB: Lactate dehydrogenase B
SIRT1: Inhibit Sirtuin 1
HMGB1: High-mobility group box 1 protein
H3K14la: Histone H3 lysine 14
PFKFB3: 6-Phosphofructokinase/fructose-2,6-bisphosphatase 3
ROS: Reactive oxygen species
